# Expert Event Segmentation of Dance Is Genre-Specific and Primes Verbal Memory

**DOI:** 10.3390/vision4030035

**Published:** 2020-08-10

**Authors:** Paula M. Di Nota, Michael P. Olshansky, Joseph F.X. DeSouza

**Affiliations:** 1 Department of Psychology, York University, Toronto, ON M3J 1P3, Canada; michael.p.olshansky@gmail.com; 2 Centre for Vision Research, York University, Toronto, ON M3J 1P3, Canada; 3 Neuroscience Graduate Diploma Program, York University, Toronto, ON M3J 1P3, Canada; 4 Graduate Program in Interdisciplinary Studies, York University, Toronto, ON M3J 1P3, Canada

**Keywords:** event segmentation, action observation, visuomotor processing, cognition, verbal memory, expertise, priming, dance

## Abstract

By chunking continuous streams of action into ordered, discrete, and meaningful units, event segmentation facilitates motor learning. While expertise in the observed repertoire reduces the frequency of event borders, generalization of this effect to unfamiliar genres of dance and among other sensorimotor experts (musicians, athletes) remains unknown, and was the first aim of this study. Due to significant overlap in visuomotor, language, and memory processing brain networks, the second aim of this study was to investigate whether visually priming expert motor schemas improves memory for words related to one’s expertise. A total of 112 participants in six groups (ballet, Bharatanatyam, and “other” dancers, athletes, musicians, and non-experts) segmented a ballet dance, a Bharatanatyam dance, and a non-dance control sequence. To test verbal memory, participants performed a retrieval-induced forgetting task between segmentation blocks. Dance, instrument, and sport word categories were included to probe the second study aim. Results of the event segmentation paradigm clarify that previously-established expert segmentation effects are specific to familiar genres of dance, and do not transfer between different types of experts or to non-dance sequences. Greater recall of dance category words among ballet and Bharatanatyam dancers provides novel evidence for improved verbal memory primed by activating familiar sensorimotor representations.

## 1. Introduction

Human observers unconsciously break down continuous streams of action into discrete chunks or events [1,2,3]. Such ‘event segmentation’ involves several cognitive functions, including attention to individual movement features and working memory (WM) to group component movements into meaningful chunks. By processing the kinematic, linguistic, and cognitive aspects of movement, event segmentation facilitates action understanding, encoding, and embodiment [4,5]. Accordingly, each type of information is processed within a distributed network of brain regions collectively referred to as a knowledge schema [6,7]. Knowledge schemas enable adaptive motor learning and accurate outcome prediction in familiar contexts [8]. The overarching goal of the present study was to determine whether priming knowledge schemas by observing familiar action sequences improved cognitive performance relative to unfamiliar experts or novices. Using a combined paradigm, the specific aims of the current study were to investigate (1) genre-specific attentional performance during an event segmentation task and (2) domain-specific (i.e., dance, music, or sports) verbal WM performance during a retrieval-induced forgetting (RIF) task. 

Attention can be measured by event segmentation, as event borders are defined by meaningful, reliable, and ecologically-relevant features of action as it unfolds in real-time. Event borders in observed action are triggered by changes in the goals, interactions, and temporal relations of observed agents [9,10]. With embodied experience, event borders can be predicted as knowledge schemas are refined [2,11]. Research on individuals with Alzheimer’s disease (AD) has shown that aberrant segmentation of a video (packing a lunchbox) predicted poorer motor performance and episodic memory of the same task [12,13]. These findings suggest that the discrete abilities to segment, describe, and encode action into memory rely on common underlying knowledge schemas, and are degraded in clinical samples. 

The chunking of component movements that can be recombined to form a novel sequence is perhaps best exemplified by dance, which has garnered a growing body of literature in the field of cognitive neuroscience [14]. While segmentation has been demonstrated during passive listening to music [15], only three studies have explored event segmentation of dance: Noble et al. [16] identified the brain regions activated at event borders of a Bharatanatyam dance in non-experts. Bläsing [17] found evidence for expert segmentation effects such that contemporary dancers identified fewer event borders for a familiar dance relative to non-dancers. Further, experts segmented the same dance sequence even less after physically embodying, learning, and performing it. Levine and colleagues [18] replicated these findings among expert figure skaters, and clarified that visual familiarity among novice observers does not replicate the effects of physical expertise. 

While these investigations compared event segmentation in non-experts and/or experts highly familiar with the genre of the experimental stimulus, it remains to be observed whether these effects are generalizable to (1) expert dancers in other genres of dance and/or (2) experts in other physical domains, including musicians and athletes. Transfer of visuomotor pattern recognition across genres or domains has been demonstrated in expert team ball sport athletes (i.e., netball, basketball, and field hockey) [19]. Therefore, Aim 1 of the present study will explore genre-specific and cross-domain event segmentation behaviors. Experts in dance, music, and sports, as well as non-experts, segmented two dance videos (ballet and Bharatanatyam) as well as a non-dance control sequence. We hypothesized that dancers familiar with the observed genre (i.e., ballet dancers segmenting a ballet video) would indicate fewer event borders than dancers from unfamiliar genres (e.g., contemporary or jazz) (Hypothesis 1; H1). We also predicted that all sensorimotor experts (dancers, athletes, and musicians) would demonstrate fewer event borders for all video conditions compared to non-experts (Hypothesis 2; H2), demonstrating generalized expertise effects during event segmentation of dance and non-dance movements.

The translational cognitive benefits of physical expertise in video gaming, sports, music, and dance, have been studied extensively [20]. Long-term physical training, especially in dance, have been shown to refine knowledge schemas through neuroplastic changes to visuomotor and cognitive processing areas [21,22,23,24,25,26,27,28]. These effects are suggested to enhance processing of, and access to, declarative and procedural information to achieve peak performance in sport as well as in laboratory tests of cognitive function [29]. Recent investigations have revealed that both observational and physical learning facilitate action verb processing [30], with prior knowledge exerting top-down cognitive influences on event segmentation behaviors and verbal memory in the same experiment [31]. We conducted a similar dual-paradigm experiment to assess Aim 2 (transfer of sensorimotor expertise to improved verbal WM performance) with the RIF task. Subjects were presented with several category–exemplar word pairs (e.g., FRUIT—apple, RELATIVE—cousin) before recalling a subset of exemplars and categories (e.g., FRUIT—ap__). This results in three types of items: practiced words from practiced categories (Rp+), non-practiced words from practiced categories (Rp−), and non-practiced words from non-practiced categories (Nrp). Subsequent recall revealed two reliable effects—a practice effect (i.e., Rp+ items recalled best) and RIF (i.e., Nrp items recalled better than Rp− items) [32,33]. It is suggested that the retrieval practice of Rp+ items creates competition for limited cognitive resources and suppresses retention of Rp− items at final recall [34,35,36]. By including dance, instrument, and sport category words, Aim 2 of the present study was to explore whether expert participants in dance, music, and athletics would have improved recall of experientially-relevant words, respectively (Hypothesis 3; H3). By priming embodied knowledge schemas with observed dance stimuli, the current study would reveal putative genre- and domain-specific transference of sensorimotor expertise to improved attentional and verbal WM performance.

## 2. Materials and Methods 

### 2.1. Participants

Based on sample sizes of precedent investigations [17] we aimed to test *n* = 20 participants in each of six groups that would test for possible familiarity effects for the event segmentation (ballet, Bharatanatyam, other genres of dance) and RIF tasks (musicians, athletes), as well as a non-expert control group for a goal *n* = 120. Sample size estimates in G*Power for a repeated measures within-between study with effect size *f* = 0.25 (which corresponds to a Cohen’s medium effect *d* = 0.05), power = 0.80, number of groups = six, number of measures = three video conditions indicated that a total sample size of *n* = 54 was required, which we exceeded with the current sample.

A total of 116 participants (89 female, mean age = 21.64 years, *SD* = 7.1) separate from those included in the validation experiment (see Supplementary File 1) were recruited from the York University community and Undergraduate Research Participant Pool (URPP), and were compensated with partial course credit. Participants were assigned to one of six groups based on self-reported previous experience—ballet, Bharatanatyam, dance (i.e., in genres other than ballet and Bharatanatyam), musician, athlete, or non-expert. Participants were deemed experts if they had a minimum of five years of experience and had practiced their skill on a regular basis (minimum three times a week) within the last two years. Many participants had experience in multiple activities (i.e., ballet, music, and sports), and were assigned to the group that they had the most experience in and/or self-reported as being their most practiced and mastered activity. Participants with dance experience in Bhangra and/or Bollywood were assigned to the dance group, as these genres are distinct from the South Asian/Tamil Bharatanatyam dance presented in our stimuli. Musician and athlete groups were included and differentiated from a general (non-dance) expert group to explore any putative and distinct contributions of musical and non-musical physical expertise, respectively. All participants had normal or corrected vision and were free of any neurological disorders. One subject from the ballet group was excluded due to medical reasons, and three participants (one dancer, one athlete, and one non-expert) were excluded following group-level outlier analyses (*z* > 2.5) on the average number of event borders. Thus, a total of 112 participants (88 female, mean age = 21.63 years, *SD* = 7.1, mean years of experience = 9.85, *SD* = 5.8) were included in analyses (Table 1). All procedures, including the validation and main experiments, were approved by the York University Human Participants Research Review Sub-Committee (Certificate # 2013-313).

### 2.2. Event Segmentation Procedure, and Stimuli

Initial briefing, instructions, and practice trial procedures were identical in the validation and main event segmentation experiments, but the number of trials differed (see Supplementary File 1). Prior to briefing, participants provided informed consent and filled out a demographic questionnaire where they self-reported the type and number of years of experience in dance or any other physical craft. These responses determined which group participants would be assigned to (N.B.: all participants in the validation experiment were non-experts).

Event segmentation was defined and explained to participants, who were instructed to watch a series of three one-minute videos (one ballet, one Bharatanatyam, and one acting) which were selected from the validation experiment (see Supplementary File 1) and originally from MP4 video files obtained from the Perception Action and Cognition Lab website (https://paco.psy.gla.ac.uk/?portfolio_page=video). Detailed description of the movement features and musical accompaniment of the videos can be found elsewhere [37]. Video clips were sampled and created using iMovie 2011 (Version 9.0.9 1795, Apple Inc., Cupertino, USA) and exported as M4V files with a 25fps frame rate and size of 480 × 272 pixels. It was ensured that all clips had similar sound profiles according to visual inspection of built-in waveforms provided by the movie editing software, and that no clip began or ended in the middle of a movement to reduce the potential of response errors or ‘misses’. To be compatible with the presentation software (MatLab, Version 7.10.0.499, The MathWorks, Inc., Natick, MA, USA and Psychtoolbox, Version 3) [38], video clips were converted to MPG format with Media Converter 2013 (Version 8, ArcSoft Inc., Fremont, CA, USA) and specified to remain at 25fps frame rate and 480 × 272 pixels. All stimuli and experimental protocols were presented using custom Matlab code (which can be obtained from the corresponding author upon request) on a 24-inch iMac desktop computer with adjusted 1024 × 640 resolution, resulting in 9.5 × 5.5 inch video playback (horizontal visual angle = 21.1°). Participants were provided with noise cancelling headphones (Model MDR-NC7, Sony Corp., Tokyo, Japan) to hear accompanying musical stimuli, with volume manually adjusted to each participant’s comfort level before the onset of the experiment. 

Participants were instructed to press the spacebar anytime they perceived one natural and meaningful unit of expressive movement begin, and another end [2,3,39]. It was also explicitly stated that participants did not need to press the spacebar once to indicate the end of a movement and then once again immediately following to indicate the start of a subsequent movement, but that one button press was sufficient to mark the “border” between two movements. Participants were reassured that there was no right or wrong answer, and to simply do their best for each trial. They were also instructed not to worry about replicating responses on previous trials for the same video but to simply segment each video as it comes, and to focus on the movements and not the background music when identifying event borders. 

**Figure 1:**
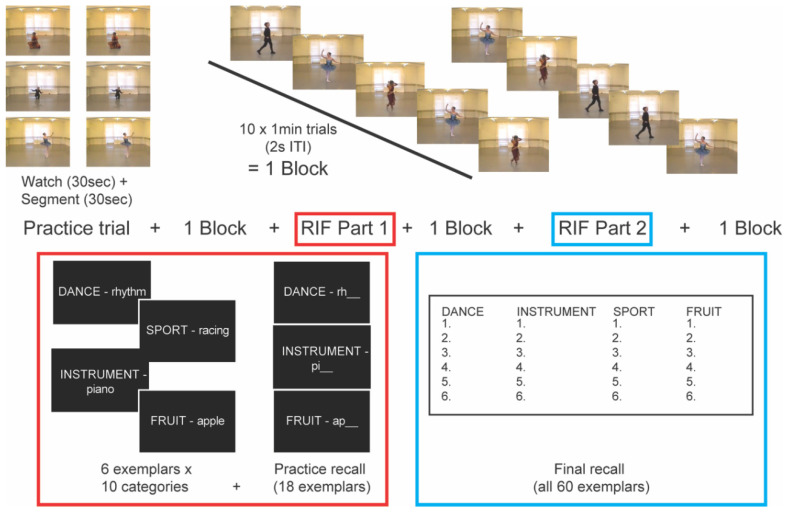
Event segmentation and retrieval-induced forgetting protocols. Based on results of the validation experiment (see Supplementary File 1), a one-minute video clip from each category (ballet, Bharatanatyam, and acting) was chosen for the event segmentation portion of the experiment, which included an interleaved retrieval-induced forgetting (RIF) memory task. Following a practice trial (**top left panel**), each video was played 10 times in random order across 3 blocks of 10 trials each. Between blocks of event segmentation, participants performed the two-part RIF task: Part 1 (**red box**) included presentation of 60 category-exemplar word pairs (10 categories × 6 words each) and practice recall of 18 exemplars. The second block of event segmentation, Part 2 (**blue box**) had participants recall as many words as possible from the original 60 category–exemplar pairs.

Following these instructions, participants were given the opportunity to perform a practice trial (Figure 1) during which the experimenter remained in the room to ensure participants understood the task and could perform it properly. Following a series of instruction screens that repeated the experimenter’s verbal instructions, a 30-s version of one of the original nine video clips used in the validation experiment was randomly selected and played twice. For the first presentation, participants were instructed not to press the spacebar but to passively watch the video in order to familiarize themselves with the scene and setting, and to be able to anticipate how to respond in the subsequent presentation. Next, participants were instructed that the same clip would play again and that they should now practice segmenting by pressing the spacebar. The experimenter observed all participants perform this practice trial, and none of them demonstrated or indicated any difficulty in performing the task. Following the practice trial, the experimenter left the testing room and continued to monitor participants for compliance through a two-way mirrored window in the adjacent room. 

Each video (ballet, Bharatanatyam, and acting) was presented ten times for a total of 30 trials, which were presented in blocks of 10 with a 2-s inter-trial interval (ITI, Figure 1). The order of the 30 video trials was randomized to minimize practice effects and encourage stimulus-driven event segmentation of all clips. Participants were told that they would perform a two-part memory task between blocks (see next section). 

### 2.3. Retrieval-Induced Forgetting (RIF) Task

#### 2.3.1. Participants

Of the 113 event segmentation participants, one (non-expert) was excluded for skipping the practice recall of Part 1, four subjects (three non-experts and one dancer) were excluded for completing Part 2 at the wrong time during the experiment, and two subjects (one athlete, one non-expert) did not complete Part 2. Four additional non-experts that were not included in the event segmentation portion of the experiment were tested to even out the group sizes (Table 1). Thus, a total sample of 106 participants (82 female, mean age = 21.56, *SD* = 7.3) were analyzed for the RIF portion of the experiment.

#### 2.3.2. Procedure

Following initial instructions on the event segmentation task, the experimenter told participants that their memory would also be assessed between blocks of event segmentation. Participants were told that they would be presented with 60 category–word pairs (see Supplementary File 2): the first word in each pair would be a category and shown in capital letters (e.g., CLOTHING), and the second word in each pair would be an exemplar from the category shown in lower case (e.g., pants). Participants were instructed to focus on the second word in each pair (i.e., the exemplar) as they would be required to remember them later in the experiment. 

Category–exemplar pairs, RIF instructions, and procedures were modeled according to [32], programmed in custom Matlab script (available from the corresponding author upon request), and integrated with the event segmentation paradigm. Word categories included “Colors”, “Fruits”, “Relatives”, “Tools”, “Weather”, “Vegetables”, and “Vehicles”, as well as “Instrument” and “Sport” words that would assess recall of experientially-relevant words in musicians and athletes, respectively (see Supplementary File 2 for full exemplar list). To test experientially-primed recall among dancers in ballet, Bharatanatyam, and dance groups, the category of “Furniture” words was replaced with “Dance” words generated by the experimenters. Exemplars were obtained by searching for synonyms of the word “dance” on three vocabulary and thesaurus websites (https://myvocabulary.com, http://theseaurus.com, and www.onelook.com). The final six exemplars were chosen by cross-referencing the results of the web searches and excluding non-English words related to ballet (e.g., “ballet”, “barre”, “plie”, or “pointe”). The final six dance exemplars were “choreography”, “jig”, “rhythm”, “tango”, “tap”, and “waltz”.

After the first block of event segmentation (Figure 1), participants performed Part 1 of the RIF task. Instructions were repeated on screen and participants were instructed to press the spacebar when they were ready to see the category–exemplar pairs. Each of the 60 pairs were presented in random order at the center of the black screen in white font (vertical visual angle = 5.6°) for 1.5 s each. After the final word pair, an instruction appeared on the screen informing participants to wait for the experimenter before proceeding. Keeping track of participants’ progress through a two-way mirror, the experimenter entered the testing room and performed a practice recall of 18 words that had just been presented (for the list of practiced words, see Supplementary File 2). Participants were shown a category and the first two letters of an exemplar that had just been shown (e.g., “FRUIT–ap_____”) and were instructed to say the word out loud if they remembered it (“apple”). If they could not remember the word, they were instructed to say “pass”, and the next word was presented on-screen by pressing the spacebar. 

Following the practice recall, the experimenter explained to the participant that they would now perform another block of the event segmentation task before completing Part 2 of the memory task. Participants were provided with a sheet of paper listing each of the ten categories with a blank numbered list from one to six beneath each category. Participants were instructed that Part 2 required them to write down as many words as they could recall when prompted by instructions on the screen. In accordance with RIF instructions [34,35], final recall was performed approximately ten minutes after presentation of the category–exemplar pairs, which aligned perfectly with performing one block of event segmentation. As such, participants were explicitly instructed not to begin filling in the final recall sheet until instructed to do so, and not to continue with the final block of event segmentation until they had completed the recall sheet for Part 2. 

### 2.4. Statistical Analyses

The dependent variable for the event segmentation portion of the experiment was the average number of event borders per condition, which were divided into early (trials 1 to 5) and late (trials 6 to 10) trials. Because the validation experiment failed to show similar frequency of event borders between video conditions (see Supplementary File 1), each condition was analyzed separately. Planned comparisons to test our first hypothesis included comparing each group to the familiar group for each dance condition (ballet and Bharatanatyam, respectively) using non-parametric Mann–Whitney U tests as in [17]. For the control (acting) video condition, between-group effects were explored with a non-parametric Kruskall–Wallis test. Within-group comparisons of early versus late segmentation trials were performed using non-parametric Wilcoxon signed-rank tests for non-normal data. All data organization and statistical analyses were conducted using custom code available from the corresponding authors in R (Version 3.4.1, R Development Core Team, Vienna, Austria). Due to the highly exploratory and preliminary nature of this research question, a liberal significance criteria was set at *p* < 0.05.

For the RIF portion of the current study, correct responses on the final recall sheet were coded as follows: practiced words from practiced categories (Rp+, e.g., “FRUIT–apple”), non-practiced words from practiced categories (Rp−, e.g., “FRUIT–peach”), and non-practiced words from non-practiced categories (Nrp, e.g., “RELATIVE–cousin”). Normality assumptions for composite Rp+, Rp−, and Nrp variables were confirmed with Shapiro–Wilk tests. To confirm successful replication of well-established practice effects (i.e., improved recall of Rp+ items over Nrp items) and RIF (i.e., improved recall of Nrp over Rp–items), and explore any between-group effects, we conducted two separate two (items) × six (groups) repeated measures ANOVAs. Overall practice recall accuracy (in percent) was explored between groups with a one-way ANOVA.

To test our second hypothesis that experts would demonstrate improved recall for experientially-relevant words, practice recall was assessed with a two (category—“Dance”, “Instrument”) × six (groups) repeated measures ANOVA, and final recall was assessed with a three (category—“Dance”, “Instrument”, “Sport”) × six (groups) repeated measures ANOVA. Statistical analyses for the RIF data were conducted in SPSS (Version 22, IBM Corp., Aramonk, USA). All pairwise comparisons were adjusted with a Bonferroni correction. Effect sizes are reported with partial eta squared and Bayes factor (BF) as a measure of the likelihood in support of the null (BF_01_) alternate (BF_10_) hypotheses using the ‘BayesFactor’ package in R (Version 0.9.12-4.2). Significance criteria for main and interaction effects was set at *p* < 0.05, and pairwise comparisons at *p_Bonf_* < 0.05.

## 3. Results

### 3.1. Event Segmentation 

#### 3.1.1. Average Event Borders

As in Bläsing [17], early (trials 1 to 5) versus late (trials 6 to 10) trials were analyzed for each condition with Wilcoxon signed-rank tests. Only ballet dancers had more segments in late trials for the ballet (*M_diff_* = 2.1, *SD_diff_* = 7.3, *W* = 37, *p* = 0.021) and acting (*M_diff_* = 1.9, *SD_diff_* = 0.49, *W* = 8.5, *p* = 0.001) conditions. No other groups differed in the average number of event borders for early and late trials in any condition. 

**Figure 2:**
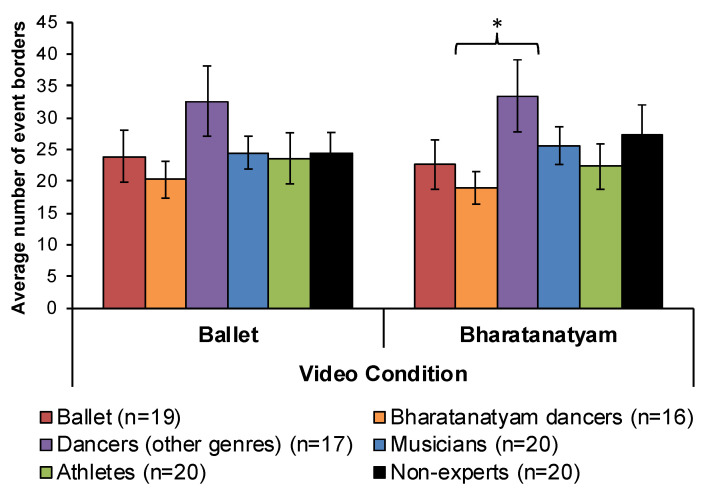
Event segmentation of dance sequences. Only during early trials did we observe genre-specific effects in Bharatanatyam dancers segmenting a familiar dance genre compared to dancers from other genres. No other between-group differences were significant. Error bars show SEM. * *p* < 0.05.

**Figure 3:**
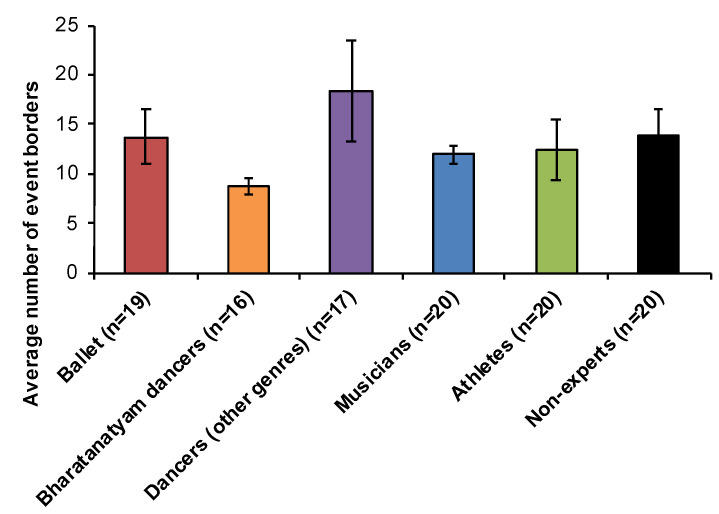
Event segmentation of a non-dance sequence. No between-group differences were observed for segmenting early or late trials of a non-dance control sequence. Mean number of event borders for all ten trials are plotted, error bars show SEM.

When comparing the familiar experts in each dance condition to all other groups, significant between-group effects were only observed during early trials. Specifically, Bharatanatyam dancers provided significantly fewer event borders when segmenting a familiar Bharatanatyam dance compared to dancers (*U* = 189.5, *p* = 0.022). However, there were no between-group differences in event segmentation of the ballet condition when comparing ballet dancers to all other groups in early or late trials (Figure 2). For the acting condition, there was no difference in the average number of event borders observed between groups for early [x**^2^** (df = 5) = 7.85, *p* > 0.10] or late trials [x**^2^** (df = 5) = 5.04, *p* > 0.10] (Figure 3). 

#### 3.1.2. Movement Features of Event Borders

To identify the most common event borders, raw event segmentation data were divided into 60 1-s bins. The frequency of button presses for each bin was determined for each group and condition. A summary of the movement features for the most-commonly-identified event borders for each condition can be found in Table 2. Across all conditions, the movement features associated with the highest incidence of event borders included changing direction of movement, raising arms, and leaping to the side for the dance conditions.

**Figure 4:**
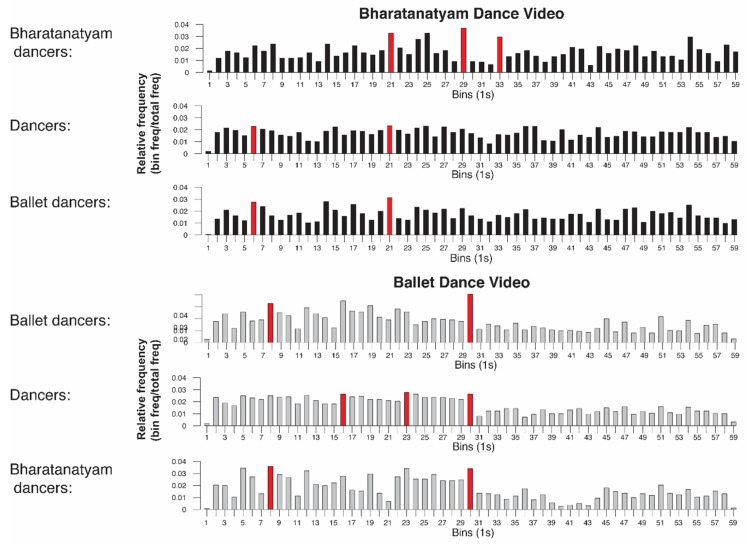
Event borders for ballet and Bharatanatyam videos among familiar and unfamiliar dancers. The most-commonly-identified event borders are marked in red. Familiar Bharatanatyam dancers identified bins 29 and 33 more frequently than other groups, while other dancers segmented bin 6. All dancers segmented the ballet video most frequently at bin 30, and unfamiliar dancers segmented bins 16 and 23.

To explore putative differences in the movement features of event borders for familiar versus unfamiliar dance sequences, we compared the most-frequently-identified bins for the dance conditions (ballet and Bharatanatyam) between the dance groups (ballet, Bharatanatyam, and dance) (Figure 4). For the Bharatanatyam condition, all groups identified bin 21 most consistently, which showed the dancer stepping backward, changing the direction of her body, raising her arm and hand, and crossing one leg over the other. Interestingly, the second-most-common border among the familiar Bharatanatyam dancers was not in the top five most-frequent bins for any other group, which involved a brief pause and arm and hand movement (bin 29). Bharatanatyam dancers also identified bowing down with the head, upper body, and arm (bin 33) more consistently and frequently than the unfamiliar dance groups. The unfamiliar ballet and dance groups also frequently identified a leap and concurrent arm movement above the head (bin 6), which was not in the top five most-frequent event borders for the Bharatanatyam dancers.

For the ballet condition, the most-commonly-identified border among familiar ballet dancers was the end of a series of four spins (bin 30), which was also in the top five most salient event borders for all other groups. While Bharatanatyam dancers were as attentive to raising of limbs with concurrent turning of the head (bin 8) as ballet dancers, dancers from other genres responded more frequently to pauses and changes in the direction and pace of movement (bins 16, 23). 

To conclude, support for genre-specific event segmentation effects (H1) was shown among Bharatanatyam dancers only (Figure 2). We found no evidence to support expert segmentation effects for non-dance sequences (H2, Figure 3). 

### 3.2. RIF Task

**Figure 5:**
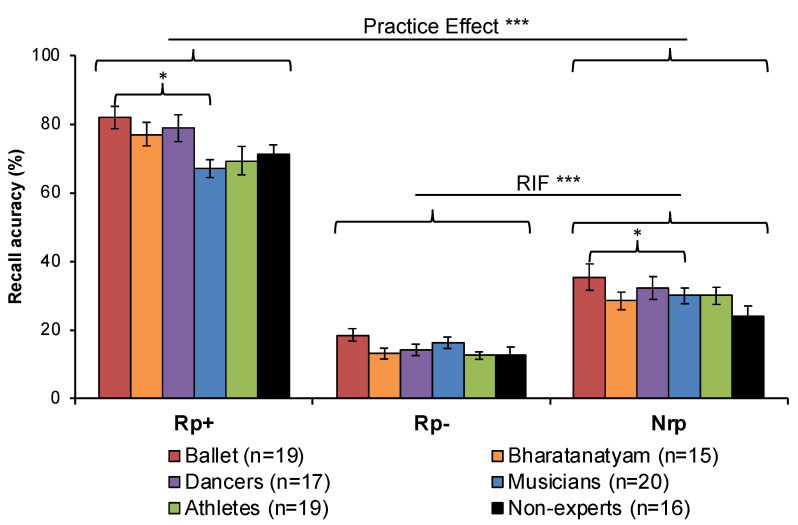
**Practice effects and RIF.** All groups showed a practice effect for improved recall of practiced words (Rp+) relative to non-practiced words from non-practiced categories (Nrp). Ballet dancers also showed better recall than musicians for Rp+ and Nrp items. All groups showed retrieval-induced forgetting, or the suppression of non-practiced words from practiced categories (Rp−) relative to non-practiced words from non-practiced categories (Nrp). Error bars show SEM. * *p* < 0.05 *** *p* < 0.001.

A significant practice effect was observed for all groups (F(1,100) = 1147.043, *p* < 0.001, η^2^ = 0.920, BF_10_ = 8.967 × 10^61^), and a significant main effect for group (F(5,100) = 3.049, *p* = 0.013, η^2^ = 0.132, BF_10_ = 3.198 × 10^−6^) revealed improved recall among ballet dancers relative to musicians (*p_Bonf_* = 0.024) (Figure 5). All groups demonstrated RIF as revealed by a significant main effect of item (F(1,100) = 230.910, *p* < 0.001, η^2^ = 0.698, BF_10_ = 1.589 x 10^23^) (Figure 5), with no other significant main or interaction effects. Groups did not differ in overall practice recall accuracy (in percent) (F(5,100) = 1.444, *p* = 0.215, BF_01_ = 0.243). Practice recall of experientially-primed words (dance and instrument categories only) showed that instrument words were remembered better than dance words among all participants (category: F(1,100) = 57.137, *p* < 0.001, η^2^ = 0.364, BF_10_ = 1.687 × 10^8^). A main effect of group approached significance (F(5,100) = 2.268, *p* = 0.053, η^2^ = 0.102, BF_01_ = 1.835 × 10^−3^) but no pairwise comparisons reached significance criteria. 

**Figure 6:**
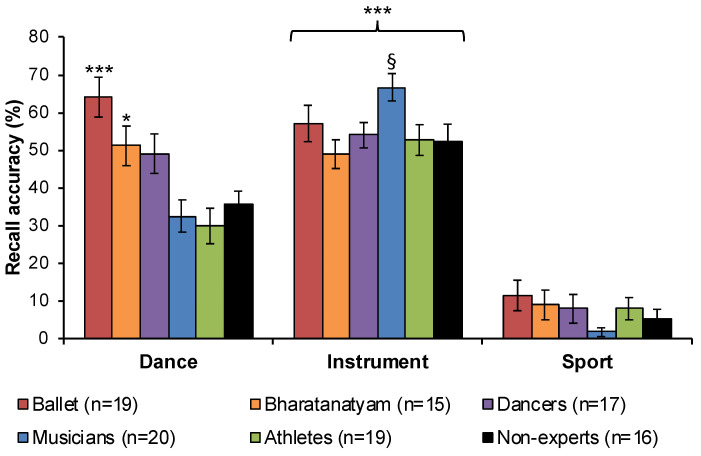
**Final recall of experientially-primed words**. Ballet and Bharatanatyam dancers showed preferential recall of experientially-primed dance words relative to non-dance experts as well as non-experts. All groups recalled instrument words more than dance and sport words. Error bars show SEM. ^§^*p* < 0.05 uncorrected * *p* < 0.05 *** *p* < 0.001.

To assess transference of physical expertise to verbal memory following priming by a visual stimulus, final recall analysis of experientially-primed words reveal a significant main effect of category (F(2,200) = 289.933, *p* < 0.001, η^2^ = 0.744, BF_10_ = 2.181 × 10^53^), demonstrating that instrument words were recalled the most and sport words were recalled the least across all groups (*p_Bonf_* < 0.001) (Figure 6). A significant main effect of group (F(5,100) = 3.371, *p* = 0.007, η^2^ = 0.144, BF_10_ = 2.167 × 10^−6^) also revealed improved recall among ballet dancers relative to athletes (*p_Bonf_* = 0.007) and non-experts (*p_Bonf_* = 0.023), but a significant category x group interaction (F(10,200) = 6.058, *p* < 0.001, η^2^ = 0.232, BF_10_ = 5.524 × 10^59^)] revealed that ballet dancers recalled more dance words than musicians, athletes (*p_Bonf_* < 0.001), and non-experts (*p_Bonf_* = 0.001), and Bharatanatyam dancers recalled more dance words than athletes (*p_Bonf_* = 0.043). At uncorrected significance levels, musicians recalled more instrument words than all other groups except for ballet dancers (Bharatanatyam: *p* = 0.003, dancers: *p* = 0.029, athletes: *p* = 0.013, non-experts: *p* = 0.014). These findings demonstrate improved recall of experientially-primed words for ballet and Bharatanatyam dancers, as well as for musicians at uncorrected significance levels, but not among athletes for sport category words (Figure 6). 

## 4. Discussion

The present investigation successfully replicated and clarified previous findings for a reduction in the number of event borders for familiar dance genres only in Bharatanatyam dancers (H1, Figure 2). Further, this pattern of expert segmentation did not extend to non-dance movement sequences (Figure 3), or to experts in other physical activities including athletics and music (H2). For the first time, the present results demonstrate transfer of sensorimotor expertise across cognitive functions. Observing a familiar dance primes improved verbal WM and recall of dance words in ballet and Bharatanatyam dancers, near-significant improvement in recall of instrument words among musicians (Figure 6), but no cross-domain transference effects were observed for athletes (H3). Acquiring sensorimotor expertise requires combining information, both specific and abstract, related to the skill through repeated and prolonged practice. What results is a specialized knowledge schema that includes a genre-specific motor repertoire, vocabulary to define, describe, and identify these movements, and memory structures to plan and anticipate movement outcomes. The current findings support transference of sensorimotor expertise to improved attentional and verbal WM performance for stimuli related to one’s expertise.

Unlike other movements that have been investigated in event segmentation paradigms, dance is not goal- or object-directed and is usually accompanied by music. Event borders in musical compositions can be detected in both trained and untrained musicians, and are differentiated by changes in tone, tempo, rhythm, pitch, and boundary silences [40]. Consistent with previously-identified movement event boundaries [1,41,42,43], changes in the direction of movement, pose, or position, relative location, speed, and acceleration were the most-commonly-identified event borders among all participants in the current study (Figure 4, Table 2). Observing both novel and learned action elicits activation from a distributed visuomotor network [21,24,44,45,46], including the frontal eye field [3], human MT+ complex [9,39], and posterior superior temporal sulcus [47], and is most active at event borders. Beyond perception of motion dynamics, visuomotor networks also involve memory regions like the parahippocampal cortex to facilitate prediction of succeeding steps [9]. Consistent with previous behavioral [17] and computational research [48,49], uncertainty can explain the observed increase in segmentation behavior among unfamiliar experts (H1, Figure 2 and Figure 4). Unfamiliar observers, who do not have embodied motor representations to rely on, face unpredictability and uncertainty of what steps should come next. Indeed, memory of events is impaired when there are more sub-events to recall [50]. When recruited together and consolidated over many years of experience and training, verbal and motor information become integrated into expert knowledge schemas. Within the context of this neuroimaging literature, the current findings provide a putative neural basis for the genre-specific consolidation of familiar dance movements (H1), and improved recall of domain-specific verbal WM following observation of familiar, embodied dance stimuli (H3).

While there is mounting neuroscientific evidence for reorganization of sensorimotor areas with dance expertise, the majority of research on functional transference between motoric and cognitive domains comes from experts in athletics, chess, computer programming, meditation, and medicine [19,29,51,52,53]. Transfer of physical expertise to improvements in verbal working and long-term memory may also be sensitive to critical periods in development [54,55,56,57]. Domain-specificity of improved speech segmentation abilities has been observed among children trained in music but not visual arts programs [58]. The closest connection between transference of dance training to improved verbal WM comes from a study on figure skaters. Relative to novices, experts showed improved motor performance and access to domain-specific vocabulary in semantic memory [59]. This may be due to different retrieval strategies such that experts rely on embodied motor processes and procedural memory while novices recruit more declarative memory [59,60,61]. Differential activation of expert versus novice knowledge schemas is supported by neuroimaging evidence that shows greater motor cortex activation among expert hockey players when listening to action sequences with hockey-related words relative to non-experts [62]. More recently, Beauprez and colleagues [30] have shown a direct link between motor, language, and memory such that physical and observational learning of unusual action sequences primes action verb processing. The current results suggest that verbal WM is primed by sensorimotor activation of highly familiar stimuli, as recall of dance-related words were significantly greater among ballet and Bharatanatyam dancers relative to non-dancers (H3, Figure 6). Despite impaired event segmentation among people with neurodegenerative disorders like AD [12,13], verbal memory can be significantly improved following training with sung versus spoken words [63]. Our research team has contributed to a growing body of promising evidence for improved cognitive, motor, and neurophysiological functioning in individuals with AD and Parkinson’s disease following music and dance practice [22,64]. Together with the present results, these investigations lend further credit to the therapeutic efficacy of dance and music for transferring improvements across motoric and cognitive domains.

Limitations of the current study include significant event segmentation effects in the first five of ten trials only. These findings suggest a decreased sensitivity to experimental measures over repeated trials. Especially among familiar observers, responses may have been saturated by the fifth of ten trials. Spontaneous segmentation behavior to novel stimuli may be better captured in early trials (i.e., less than five, see also [17]). The fact that familiarity effects were only observed in Bharatanatyam dancers (H1) and not ballet dancers for their familiar dance condition, or in athletes recalling sport category words (H3), could be attributed to several limitations of the experimental stimuli. Despite claiming to record three videos with a similar type, number, and pace of movements [37], the results of both the validation (Figure S1) and main event segmentation experiments (Figure 2 and Figure 3) showed that the acting video had significantly fewer event borders and movement phrases than both dance conditions. In addition, we found great variability in the number of segments across several one-minute portions of the ballet and Bharatanatyam dance during stimulus development. In fact, Jola and Grosbras [65] show evidence for more perceived gestures in the Bharatanatyam video compared to the ballet and acting videos among novice viewers, which may account for the greater number of event borders observed in unfamiliar dancers (Figure 2). Additionally, the musical accompaniment in the Bharatanatyam video features a vocal track sung in Tamil. This may have impacted participants’ ability to segment movement independent of auditory, musical, or lyrical cues despite receiving explicit instructions to focus on the movements and not the music (see Section 2.2). To dissociate the contributions of movement and music in dance perception and segmentation, future studies should include eye tracking and/or randomly and systematically present unimodal (i.e., auditory music and visual dance) and multimodal stimuli. Bläsing [17] attempted such an investigation but presented all unimodal auditory trials after 10 audiovisual trials, confounding interpretation of any uni- and multimodal effects with possible practice or learning effects.

The sport category words used in the RIF task and originally established by Battig and Montague [66] had the lowest recall of all experientially-primed words that we investigated (Figure 6) and included racing, sailing, climbing, fishing, skating, and riding. While poor recall of these items could be attributed to the fact that they were Nrp words and not included in practice recall, the well-established RIF effect that we successfully replicated (Figure 5) should have induced better recall of these words relative to non-practiced dance or instrument words. Instead, we attribute this poor performance across all groups to the nature of the items as more recreational in nature relative to more popular, commercialized or team-based sports like football, hockey, soccer or baseball that participants reported having experience with. Additionally, all other exemplars were nouns while sport words were verbs, which may have also confounded recall for these Nrp items. Future investigators utilizing this RIF word bank should consider revising and updating these items to control for participants’ expectation of more common sport exemplars.

## 5. Conclusions 

The current study provides novel evidence for transfer of expertise across motor and cognitive domains that is specific to the expert’s experience. By priming expert visuomotor networks with a familiar dance video during an event segmentation paradigm, improved verbal memory was observed during a RIF task. These findings also clarify that previously-demonstrated expert segmentation effects are specific to familiar dance genres, and not unfamiliar ones or non-dance sequences. Based on neurophysiological evidence linking motor, memory, and language areas of the brain, the current findings reflect transference of functional improvement in attention and verbal WM following prolonged training and expertise in dance. Consolidation of expert knowledge schemas bears promising implications for the efficacy of dance therapy for motor and cognitive disorders.

## Figures and Tables

**Table 1:** Demographic information for event segmentation and retrieval-induced forgetting (RIF) tasks.

Group	Event Segmentation			Retrieval-Induced Forgetting		
	*n* (Female)	Age, M (*SD*)	Years of Experience, M (*SD*)	*n* (Female)	Age, M (*SD*)	Years of Experience, M (*SD*)
Ballet	19 (19)	21.8 (7.6)	12.8 (5.1)	19 (19)	21.8 (7.6)	12.8 (5.1)
Bharatanatyam	16 (16)	20.4 (5.9)	11.7 (7.4)	15 (15)	20.5 (6.1)	12.2 (7.5)
Dance *	17 (15)	24.1 (7.5)	11.4 (5.5)	17 (14)	23.8 (7.5)	11.6 (5.4)
Musician	20 (13)	21.9 (10.1)	10.9 (4.5)	20 (13)	21.9 (10.1)	10.9 (4.5)
Athlete *	20 (12)	20.1 (3.0)	9.9 (3.4)	19 (10)	20.1 (3.0)	9.6 (3.1)
Non-Expert *	20 (13)	21.7 (7.0)	3.1 (2.2)	16 (11)	21.3 (7.6)	3.1 (2.4)
**Total**	**112 (88)**	**21.63 (7.1)**	**9.85 (5.8)**	**106 (82)**	**21.6 (7.3)**	**10.1 (5.7)**

* Denote groups that have different participant data for the event segmentation and RIF portions of the experiment.

**Table 2:** Movement features of the most-frequently-identified event borders by condition.

Condition	Bin	Movement
**Ballet**	30	Concluding a set of four spins
	8, 12	Raise arm, leg, turn head (to left and right, respectively)
	16	Change direction and pace of movement
	22, 23	Stop, change direction of movement, and begin set of four spins
	5, 10	Large step or leap to the side
**Bharatanatyam**	21	Step backward, change direction, raise hand, cross leg
	54	Pause after stepping forward, raise arm, nod head and hand
	6–8	Leap to the side while moving arm above head
	25	Change direction, step, lift arms
	33, 11	Bow down with head, upper body, and arm
**Acting**	40–43	Crouched, tap knee with hands
	45	Stop knee tapping sequence and raise arms
	5	Turn head, raise arms
	37, 55–58	Crouched, tap knee with hands
	59	Stop knee tapping sequence and raise arms

Each 60-s video was divided into 1-s bins, and the frequency of button presses was determined for each bin. The movement features of the top five bins for each condition are shown here (collapsed across groups).

## Supplementary Materials

The following are available online at https://www.mdpi.com/2411-5150/4/3/35/s1, Supplementary File 1: Stimulus Validation Experiment, Supplementary File 2: Category–Exemplar Word Pairs Presented During the RIF Task, Figure S1: Results of stimulus validation study.
