# Supplementary material for: Expert Event Segmentation of Dance Is Genre-Specific and Primes Verbal Memory
Source: Vision (Basel). 2020 Aug 10;4(3):35. doi: 10.3390/vision4030035 (PMC7559184; doi:10.3390/vision4030035)
Supplement: Supplementary file 1 [file vision-04-00035-s001.zip › Supplementary File 1.docx]

**Supplementary File 1: Stimulus Validation Experiment**

We performed a Validation Experiment with a separate group of non-expert participants for two reasons: 1) to identify one video clip from each of the three conditions (ballet dance, Bharatanatyam dance, non-dance) that contained equal numbers of event borders in order to attribute any observed between- or within-group differences to participants’ prior experience, and 2) to determine the parameters (i.e., trial, block, and experiment duration) that would optimize the number of segmentation trials per condition while including an interleaved memory task and minimizing overall experiment duration and possible fatigue.

**Participants**

A total of 30 participants (20 female, mean age = 23.3 years, *SD* = 4.5) were recruited from the York University community and Undergraduate Research Participant Pool (URPP), and were compensated with partial course credit. One participant’s data was excluded due to non-compliance, and another two subjects were excluded following outlier analyses (i.e., for having normalized event borders exceeding 2.5 standard deviations from the mean of all participants, *z* > 2.5). Thus, a total of 27 participants (18 female, mean age = 23.4 years, *SD* = 4.5) were included in final analyses, and reported an average of 2.46 (*SD* = 3.5) years of experience in either dance, sports, or music. To reduce any possible expertise effects, we exclusively recruited non-experts for the Validation Experiment. Participants were deemed experts if they had a minimum of 5 years of experience, and had practiced their skill on a regular basis (minimum 3 times a week) within the last two years. No participants in the Validation Experiment met this criteria, and those with more than 5 years’ experience discontinued regular practice more than two years prior to the study. All participants had normal or corrected vision and were free of any neurological disorders. All procedures, including the validation and main experiments, were approved by the York University Human Participants Research Review Sub-Committee (Certificate # 2013-313).

**Video Stimuli and Apparatus**

Three one-minute video clips were selected and saved from each of the original Ballet (4mins 53sec), Bharatanatyam (5min 5sec), and Acting (4min 46sec) MP4 video files obtained from the Perception Action and Cognition Lab website (<http://paco.psy.gla.ac.uk/index.php/component/content/article/39-res/res-proj/67-watching-dance-kinesthetic-empathy>). Detailed description of the movement features and musical accompaniment of the videos can be found elsewhere [37]. The nine clips were sampled using iMovie 2011 (Version 9.0.9 1795, Apple Inc.). It was ensured that all clips had similar sound profiles according to visual inspection of built-in waveforms provided by the movie editing software, and that no clip began or ended in the middle of a movement to reduce the potential of response errors or ‘misses’. Each of the resulting nine clips were exported as M4V files with a 25fps frame rate and size of 480 x 272 pixels. To be compatible with the presentation software (MatLab, Version 7.10.0.499, The MathWorks, Inc., Natick, MA, and Psychtoolbox, Version 3) [38], video clips were converted to MPG format with Media Converter 2013 (Version 8, ArcSoft Inc., Fremont, California) and specified to remain at 25fps frame rate and 480 x 272 pixels. All stimuli and experimental protocols were presented using custom Matlab code on a 24-inch iMac desktop computer with adjusted 1024 x 640 resolution, resulting in 9.5 x 5.5 inch video playback (horizontal visual angle = 21.1**°**). Participants were provided with noise cancelling headphones (Model MDR-NC7, Sony Corp., Tokyo) to hear accompanying musical stimuli, with volume manually adjusted to each participant’s comfort level before the onset of the experiment.

**Procedure and Statistical Analyses**

Prior to briefing, participants provided informed consent and filled out a demographic questionnaire where they self-reported the type and number of years of experience in dance or any other physical craft. These responses determined which group participants would be assigned to (all non-experts for Validation Experiment). Event segmentation was defined and explained to participants, who were instructed to watch a series of nine one-minute videos (3 x ballet, 3 x Bharatanatyam, 3 x acting) that were played five times each for a total of 45 trials. Participants were instructed to press the spacebar anytime they perceived one natural and meaningful unit of expressive movement begin, and another end [2,3,39]. It was also explicitly stated that participants did not need to press the spacebar once to indicate the end of a movement and then once again immediately following to indicate the start of a subsequent movement, but that one button press was sufficient to mark the “border” between two movements. Participants were reassured that there was no right or wrong answer, and to simply do their best for each trial. They were also instructed not to worry about replicating responses on previous trials for the same video but to simply segment each video as it comes, and to focus on the movements and not the background music when identifying event borders.

Following these instructions, participants were given the opportunity to perform a practice trial during which the experimenter remained in the room to ensure participants understood the task and could perform it properly. Following a series of instruction screens that repeated the experimenter’s verbal instructions, a 30-second version of one of the nine video clips was randomly selected and played twice. For the first presentation, participants were instructed not to press the spacebar but to passively watch the video in order to familiarize themselves with the scene and setting, and to be able to anticipate how to respond in the subsequent presentation. Next, participants were instructed that the same clip will play again and that they should now practice segmenting by pressing the spacebar. The experimenter observed all participants perform this practice trial, and none of them demonstrated or indicated any difficulty in performing the task. Following the practice trial, the experimenter left the testing room and continued to monitor participants for compliance through a two-way mirrored window in the adjacent room. The order of the 45 video trials was randomized to minimize practice effects and encourage stimulus-driven event segmentation of all clips. Trials were presented in four blocks of ten trials (2-second inter-trial interval) and a final shorter block of five trials. Blocks were separated with an instruction screen that indicated how many blocks were left, repeated the event segmentation instructions, and offered an optional break. Participants continued to the next block by pressing the spacebar.

The dependent variable for the Validation and Event Segmentation Experiments were the average number of event borders for each video clip across all trials, and were analyzed for normality with Shapiro-Wilk tests and subsequently compared with non-parametric related-samples Wilcoxon signed rank tests for non-normal data using SPSS (Version 22, IBM Co., Armonk, NY).

**Results**

Statistical analyses revealed that all of the Acting video clips had significantly fewer event borders than any of the Ballet and Bharatanatyam videos (Figure S1). Acting Video 3 was selected for the final Event Segmentation Experiment due to having the highest number of average event borders, and will be analyzed separately from the dance videos for possible between-group effects. While Ballet Video 1 did not significantly differ from any of the Bharatanatyam video clips, it was not selected for the final experiment due to having the highest variability in responses (*SD* = 23.3). Thus, Video 2 for the Ballet and Bharatanatyam conditions were selected for the final experiment due to having the lowest significance value between them (*p* = 0.039), but will also be analyzed separately for between-group effects.


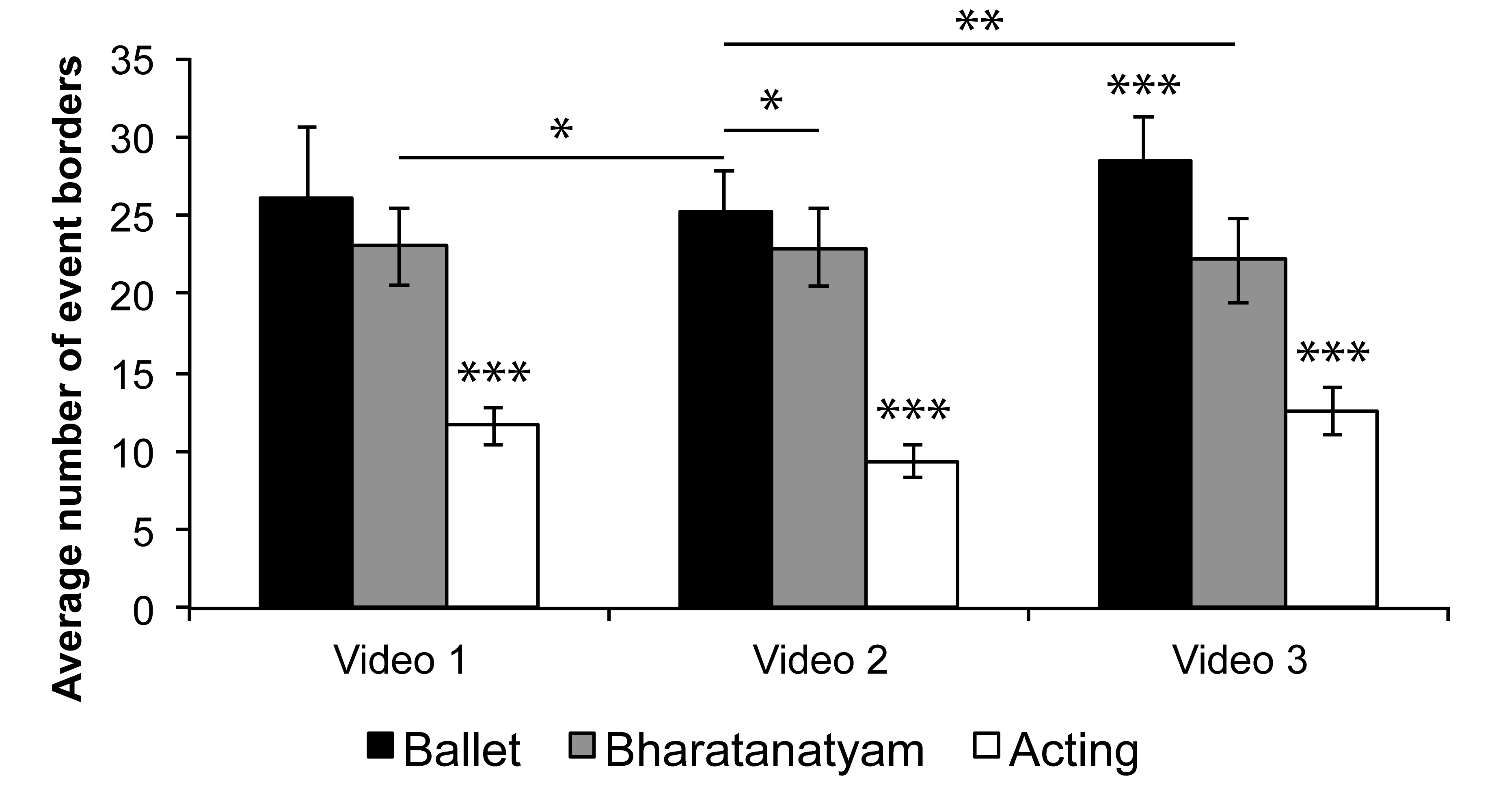


**Figure S1. Results of stimulus validation study**. Only ballet Video 1 did not differ from any of the other dance videos, but had the highest variation in event borders. All acting videos had significantly fewer event borders than the ballet and Bharatanatyam videos.

* p<0.05 ** p <0.01 *** p <0.001. Error bars show SEM.
