# Supplementary material for: Expert Event Segmentation of Dance Is Genre-Specific and Primes Verbal Memory
Source: Vision (Basel). 2020 Aug 10;4(3):35. doi: 10.3390/vision4030035 (PMC7559184; doi:10.3390/vision4030035)
Supplement: Supplementary file 1 [file vision-04-00035-s001.zip › Supplementary File 2.docx]

Supplementary File 2 - Category-Exemplar Word Pairs Presented During the RIF Task.

A ‘1’ indicates the designation of each pair as a practiced word from a practiced category (Rp+), non-practiced word from a practiced category (Rp-), or a non-practiced word from a non-practiced category (Nrp).

| **Word** | **Practiced word, practiced category: Rp+** | **Non-practiced word, practiced category: Rp-** | **Non-Practiced word, non-practiced category: Nrp** |
| --- | --- | --- | --- |
| COLOUR - black |  | 1 |  |
| COLOUR - blue |  | 1 |  |
| COLOUR - brown | 1 |  |  |
| COLOUR - green |  | 1 |  |
| COLOUR - purple | 1 |  |  |
| COLOUR - yellow | 1 |  |  |
| FRUIT - apple | 1 |  |  |
| FRUIT - banana |  | 1 |  |
| FRUIT - cherry | 1 |  |  |
| FRUIT - grape | 1 |  |  |
| FRUIT - peach |  | 1 |  |
| FRUIT - pear |  | 1 |  |
| DANCE - choreography | 1 |  |  |
| DANCE - jig | 1 |  |  |
| DANCE - rhythm | 1 | 1 |  |
| DANCE - tango |  | 1 |  |
| DANCE - tap |  |  |  |
| DANCE - waltz |  | 1 |  |
| INSTRUMENT - drum | 1 |  |  |
| INSTRUMENT - flute |  | 1 |  |
| INSTRUMENT - guitar |  | 1 |  |
| INSTRUMENT - piano | 1 |  |  |
| INSTRUMENT - trumpet |  | 1 |  |
| INSTRUMENT - violin | 1 |  |  |
| RELATIVE - cousin |  |  | 1 |
| RELATIVE - daughter |  |  | 1 |
| RELATIVE - father |  |  | 1 |
| RELATIVE - husband |  |  | 1 |
| RELATIVE - sister |  |  | 1 |
| RELATIVE - uncle |  |  | 1 |
| SPORTS - climbing |  |  | 1 |
| SPORTS - fishing |  |  | 1 |
| SPORTS - racing |  |  | 1 |
| SPORTS - riding |  |  | 1 |
| SPORTS - sailing |  |  | 1 |
| SPORTS - skating |  |  | 1 |
| TOOL - drill |  | 1 |  |
| TOOL - hammer |  | 1 |  |
| TOOL - ladder | 1 |  |  |
| TOOL - nail | 1 |  |  |
| TOOL - ruler |  | 1 |  |
| TOOL - screw | 1 |  |  |
| VEGETABLE - beans |  |  | 1 |
| VEGETABLE - carrot |  |  | 1 |
| VEGETABLE - corn |  |  | 1 |
| VEGETABLE - lettuce |  |  | 1 |
| VEGETABLE - peas |  |  | 1 |
| VEGETABLE - potato |  |  | 1 |
| VEHICLE - bicycle | 1 |  |  |
| VEHICLE - boat |  | 1 |  |
| VEHICLE - bus | 1 |  |  |
| VEHICLE - plane | 1 |  |  |
| VEHICLE - train |  | 1 |  |
| VEHICLE - truck |  | 1 |  |
| WEATHER - clouds |  |  | 1 |
| WEATHER - rain |  |  | 1 |
| WEATHER - snow |  |  | 1 |
| WEATHER - storm |  |  | 1 |
| WEATHER - thunder |  |  | 1 |
| WEATHER - wind |  |  | 1 |
