# Supplementary figures and images for: Expert Event Segmentation of Dance Is Genre-Specific and Primes Verbal Memory
Source: Vision (Basel). 2020 Aug 10;4(3):35. doi: 10.3390/vision4030035 (PMC7559184; doi:10.3390/vision4030035)

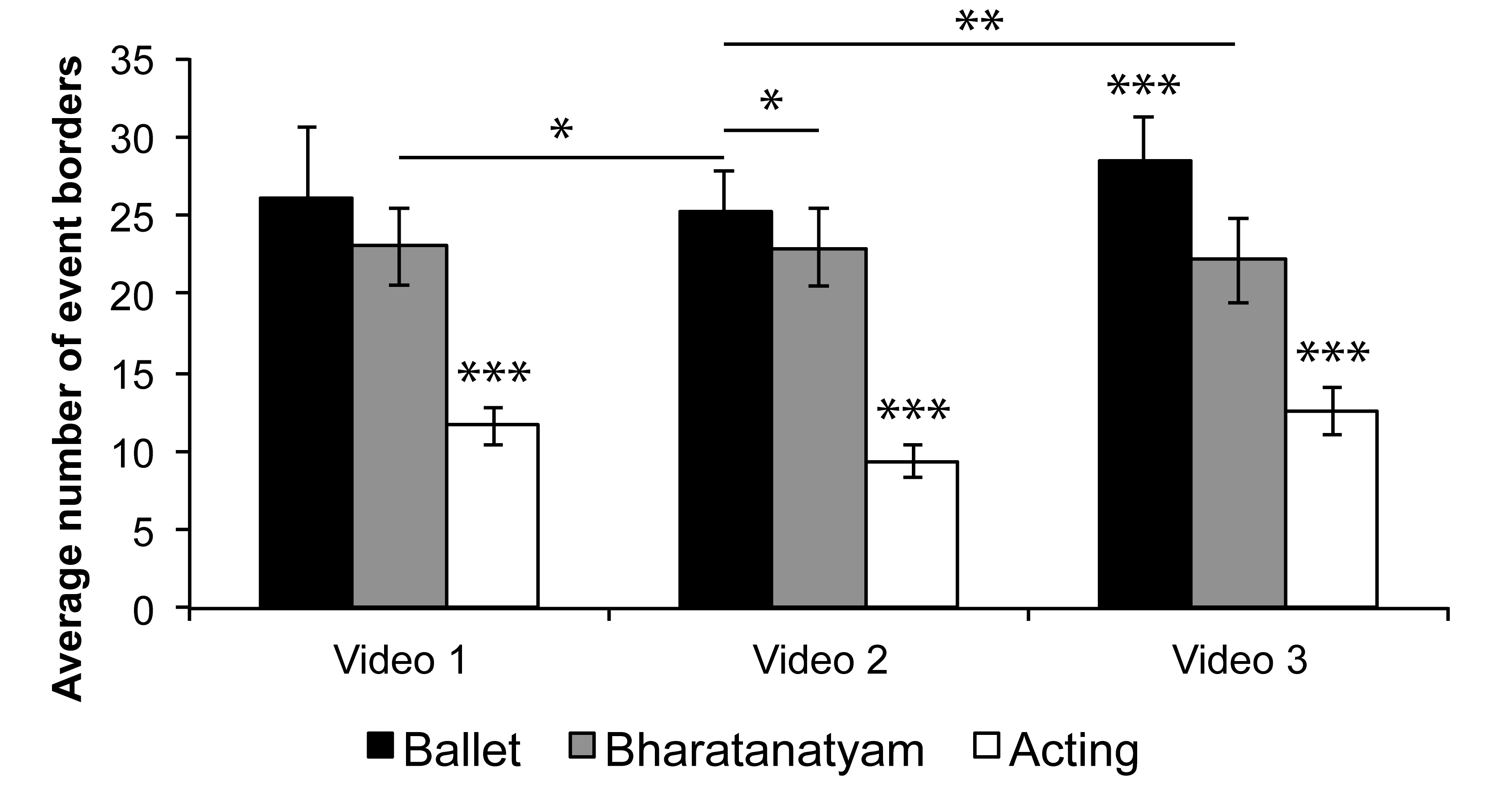

Supplement: Supplementary file 1 [file vision-04-00035-s001.zip › Figure S1.tif]
